# Determinants of public malaria awareness during the national malaria elimination programme: a cross-sectional study in rural China

**DOI:** 10.1186/s12936-016-1427-y

**Published:** 2016-07-19

**Authors:** Shangfeng Tang, Lu Ji, Tao Hu, Ghose Bishwajit, Da Feng, Hui Ming, Yue Xian, Qian Fu, Zhifei He, Hang Fu, Ruoxi Wang, Zhanchun Feng

**Affiliations:** School of Medicine and Health Management, Tongji Medical College, Huazhong University of Science & Technology, Wuhan, Hubei People’s Republic of China; Cancer Center, Sun Yat-sen University, Guangzhou, Guangdong People’s Republic of China; Bureau of Disease Prevention and Control, National Health and Family, Beijing, China; University of Nottingham, Nottingham, UK

**Keywords:** Malaria, Knowledge, Factors, Health promotion, Community media, Elimination

## Abstract

**Background:**

Public malaria health promotion is an integral part of the national malaria elimination programme, which was launched by the Chinese government in 2010. However, the public awareness of malaria needs to improve. This study aims to explore the determinants of public awareness of malaria.

**Methods:**

A cross-sectional survey was conducted using stratified sampling method from June 2015 to March 2016. Bivariate logistic regression was performed to explore the association between predictors and malaria awareness in the sample population. The homogeneity of the interaction between group assignment and the degree of knowledge related to malaria among the subgroups was calculated by Cochran–Mantel–Haenszel test.

**Results:**

Community media (including bulletin boards of village clinics or township hospitals, newspapers, exercise books, shopping bags, aprons, disposable cups, leaflets and banner advertisements) was the most prominent determinant influencing public awareness of malaria. The probability of having high-degree of knowledge about malaria among participants who received malaria-related information from community media were 3.99 times greater than those who did not (odds ratio 3.99, 95 % confidence interval 3.04–5.25, p < 0.001). Moreover, socio-demographic predictors including age, distance to township hospital, endemic county type, history of suffering from malaria, electronic media, self-assessed household income level, educational attainment and the knowledge about malaria were clearly associated with public awareness of malaria.

**Conclusions:**

Community media played the most important role in public awareness of malaria. However, only a few participants have received malaria knowledge through this media. It suggests that community media was an effective publicity material, which should expand its coverage. Malaria health promotion campaign needs to be aligned with target populations, in particular, people who are under 45 years old and residents (especially in type-3 counties) in remote areas.

## Background

Malaria is a leading cause of disease burden across the world, especially in developing countries. It is responsible for approximately 3.2 billion people at risk of suffering from malaria [1]. In compliance with a strategic reorientation from an interruption of malaria transmission in the short term to a reduction of malaria burden in the long run, a large amount of malaria-endemic countries in Africa have achieved substantial reductions in malaria transmissions [2]. During the past decades, an appreciable decline in malaria prevalence has been achieved by the efforts of local public health sectors operating in conjunction with international community [e.g., the World Health Organization (WHO)] [3]. Additionally, the WHO has set an ambitious target of reducing global malaria burden by 90 % by 2030 [4]. In Asia Pacific, the Asia Pacific Malaria Elimination Network was established a collaborative relationship among 14 countries in an effort to address the challenges brought about by malaria and, therefore, eliminate the disease by 2030 [5].

Historically, China used to be a malaria-epidemic country with a peak of about 2961 cases per 100,000 population in the 1970s [6]. Thanks to various governmental anti-malarial activities, the prevalence of malaria has declined substantially in most parts of the country during the past few decades [7, 8]. In response to the advocacy of WHO for malaria elimination at the national level, the Chinese government launched a decade-long malaria elimination programme (NMEP) in 2010 targeting at eliminating indigenous malaria cases [9]. Although only a relatively small number of indigenous malaria cases have been detected annually in recent years (especially 56 cases in 2014) [10], several thousands of malaria cases have been imported into China. There is a broad consensus about a resurgence of malaria in the endemic countries due to its vectorial capacity. The imported malaria cases become the sole, or at least major, challenge the elimination of malaria [11–14]. Also, the malaria resurgence in Greece is a reminder that the threat of imported cases merits serious attention [15].

Previous studies have shown that malaria health promotion, in accordance with the local epidemiological status and practice [16, 17], is deemed as an integral component of elimination [18], not only to improve the acceptance of surveillance activities in the community level [2], such as “1-3-7″ pattern [19], but also to improve the behaviours of self-protection and treatment-seeking [20, 21]. Public awareness is the cornerstone of the realization and maintenance of the elimination programmes [2, 22]. However, the low degree of public knowledge of malaria and potential consequences remains a critical challenge for the elimination of this disease in China [23, 24]. Given the importance of public awareness to malaria elimination, Chinese Center for Disease Control and Prevention system has carried out most of the studies since 2004 [25]. However, the determinant factors for public knowledge of malaria has not been completely explored during the NMEP. Therefore, this study was conducted to explore the determinants of malaria awareness in four provinces and to help improve the malaria promotion strategies in these regions.

## Methods

### Study population and sampling

This study was based on data obtained from a cross-sectional field survey conducted in the in rural areas of Guangxi autonomous region, Chongqing municipality, Hubei and Anhui province from June 2015 to March 2016. The areas were selected due to their relatively higher degree of prevalence of malaria in history. The NMEP identified three different types of malaria endemic counties (MEC) according to their annual incidences of local malaria. Type-1 county includes those with at least 1 malaria case per year per 10,000 residents between the years 2006 and 2008. Type-2 county includes those with lower than 1 case per 10,000 residents and type-3 county refers to those areas with no malaria cases during the same years (2006–2008). A stratified multiple stage sampling procedure across MECs, townships, villages was employed. Stratification was performed by dividing the NMEPs into three types of counties each encompassing three randomly selected MECs each of which contained three randomly selected townships depending on their malaria endemic status. Each township included three villages based on the distance (<1, 1–2 and >2 km) to township hospitals. In each village,twenty villagers were selected randomly for interviews. Finally, 1321 villagers were surveyed with a response rate of 81.54 %

### Variables and measures

The interviews were conducted by trained medical postgraduates from School of Medicine and Health Management of Tongji Medical College. A structured questionnaire was used to obtain public awareness of malaria, relevant socio-demographic information and information regarding sources of receiving malaria information. The questionnaire encompassed thirteen close-ended questions (with only one correct choice) regarding causes, symptoms and prevention of malaria. Each correct answer was assigned score 1, and the total score ranged from 0 to 13. No scores were assigned for wrong and ‘do not know’ answers. The level of knowledge was dichotomized as high-degree (≥60 % correct answers) and low-degree (<60 % correct answers) [24, 26]. Though the knowledge score is a continuous variable, in this study, it was categorized as a binary variable since data were not normally distributed.

Socio-demographic factors have been reported to play an important role in determining individuals’ health knowledge [27, 28]. Hence, the potential relevant factors were included in this analysis. The socio-demographic and economic characteristics included (1) sex, (2) ethnicity, (3) age, (4) educational attainment, (5) type of occupation, (6) number of household members, (7) marital status, (8) type of county, (9) self-assessed household income and (10) distance to township hospital.

Various malaria health promotion activities were conducted to spread information regarding malaria through media, newspapers, periodicals and networks [6], and complemented by bulletin boards of village clinics or township hospitals, exercise books, shopping bags, aprons, disposable cups, leaflets and banner advertisements, which ourre found during the pre-investigation. Therefore, the sources of malaria information were classified and collected at three different levels: (11) Individual level: a history of suffering from malaria, (12) community media including bulletin boards of village clinic or township hospital, newspaper, exercise book, shopping bag, apron, disposable cup, leaflet, and banners advertisements, and (13) electronic media including television, radio, computer and internet.

### Statistical analysis

Epidata3.1 (USA) was adopted for the establishment of databases. Data were double entered, and the input errors and logic errors during data entry were revised during data screening. Data analysis were performed with Statistical Package for Social Sciences (SPSS version 13.0; Inc., USA). The characteristics of sample population presented as categorical variables were summarized using cross-tabulations. The comparisons of proportions across variables of interest between two groups (good and poor) were computed by using Chi square test. Bivariate logistic regression was performed to explore the association between predictors and public awareness of malaria. Potential explanatory predictors available with statistically significant difference (p < 0.05), were finally enrolled in this model. Wald statistic with backward stepwise selection method was used to identify statistically significant variables, and the remained variables were determined by 5 % level. Additionally, the homogeneity of the interaction between the group assignment and the rates of good knowledge across various subgroups were calculated by Cochran–Mantel–Haenszel test [29, 30]. The rates of high-degree of knowledge regarding malaria between non-exposure and exposure groups were compared by Chi square test. The odds were computed to obtain 95 % confidence intervals (95 % CI) and odds ratios (OR).

## Results

### Sample characteristics

As shown in Table 1, most of the participants were of Han ethnicity (65.5 %), farmer migrants (58.9 %), and married (87.2 %). Approximately 57.2 % of them obtained more than 6 years of education. Compared with villagers, 42.5 % of the population considered their household income low. Approximately 52.2 % of the respondents had a family of three or four members. In terms of media, they used to receive information regarding malaria, 33.7 % of villagers received malaria knowledge through community media. 23.8 % of them were aware of malaria because they had a history of suffering from malaria. The rate of electronic material spreading malaria knowledge was the lowest among the listed instruments, 22.9 % of them use it to gain information regarding malaria.

**Table 1:** Factors associated with public malaria awareness with univariate analyses

Characteristics	n	%	Low degree knowledge (n = 634, %)	High degree knowledge (n = 687, %)	χ2	p
Socio-demographic characteristics						
Sex					5.7	0.02
Male	643	48.7	45.3	51.8		
Female	678	51.3	54.7	48.2		
Ethnicity					7.8	<0.01
Han	865	65.5	61.7	69.0		
Minorities	456	34.5	38.3	31.0		
Age					19.1	<0.01
<29	288	21.8	26.4	17.4		
30–44	325	24.6	25.2	24.1		
45–59	394	29.8	26.8	32.7		
>60	314	23.8	21.6	25.8		
Education attainment					6.2	0.05
<7 years	566	42.9	44.6	41.2		
7–9 years study	517	39.1	40.1	38.3		
>9 years study	238	18.0	15.3	20.5		
Type of occupation					12.7	0.01
Self-employed worker	219	16.6	15.6	17.5		
Farmer migrant	778	58.9	57.1	60.5		
Non-work	193	14.6	17.7	11.8		
Worker	52	3.9	3.0	4.8		
Student	79	6.0	6.6	5.4		
Number of household member					14.2	<0.01
<3	404	30.6	27.5	33.4		
3–4	689	52.2	51.3	52.9		
>4	228	17.2	21.2	13.7		
Martial status					2.4	0.12
No	203	15.3	17.1	13.8		
Yes	1118	84.7	82.9	86.2		
Type of county					45.7	<0.01
Type-1	404	30.6	27.6	33.3		
Type-2	515	39.0	33.1	44.4		
Type-3	402	30.4	39.3	22.3		
Self-assessment of household income level					18	<0.01
Low	560	42.4	47.1	38.0		
Middle	639	48.4	42.3	54.0		
High	122	9.2	10.6	8.0		
Distance to township hospital (km)					7.53	0.06
<1	360	27.2	23.7	30.6		
1–2	323	24.5	25.7	23.2		
2–3	235	17.8	18.6	17.1		
>3	403	30.5	32.0	29.1		
Sources of malaria information						
History of suffering from malaria					21.3	<0.01
No	1007	76.2	81.9	71.0		
Yes	314	23.8	18.1	29.0		
Community media					88.1	<0.01
No	876	66.3	79.0	54.6		
Yes	445	33.7	21.0	45.4		
Electronic media					11.1	<0.01
No	1018	77.1	81.1	73.4		
Yes	303	22.9	18.9	26.6		

### Differences in the status of knowledge regarding malaria

Table 1 shows the comparison between high-degree and low-degree groups in terms of their socio-demographic and sources of receiving malaria information. Statistically-significant differences were found among various subgroups except for marital status. The residents with high degree of malaria knowledge seemed to be males of Han ethnicity, farmer migrants between 45 and 59 years old, with 7–9 years education and no less than three household members. The residents with middle housed income living close to the township hospital in type-1 and type-2 counties are seemed more likely to have high-degree of malaria knowledge. Those who had a history of suffering from malaria and who received malaria information from community media or electronic media seemed more likely to have higher level of knowledge than those who did not.

### Predictors affecting public malaria awareness

After discovering that marital status was not associated with malaria awareness, this variable was excluded from the original regression model. Compared with high-degree knowledge group, a binary logistic regression was employed to examine the potential determinates of public malaria awareness among 1321 residents. Age, type of county, self-assessment of household income level, distance to township hospital, experience of suffering from malaria, community media, electronic media and education were finally retained in this model. More details were showed in Table 2.

**Table 2:** Outcome of multivariable analyses exploring predictors of public malaria awareness

Predictors	Reference	B	P	OR	95 % CI	
					Lower	Upper
Age	<29		<0.001			
30–44		0.48	0.014	1.61	1.10	2.36
45–59		0.86	<0.001	2.37	1.62	3.47
>60		1.22	<0.001	3.38	2.20	5.19
Type of county	Type-3		<0.001			
Type-1		0.48	0.007	1.61	1.14	2.29
Type-2		0.97	<0.001	2.63	1.94	3.56
Self-assessment of household income level	Low		<0.001			
Middle		0.58	<0.001	1.79	1.37	2.35
High		0.02	0.932	1.02	0.65	1.61
Distance to township hospital (km)	<1		0.008			
1–2		−0.59	0.001	0.55	0.39	0.79
2–3		−0.43	0.027	0.65	0.45	0.95
>3		−0.38	0.024	0.68	0.49	0.95
History of suffering from malaria	No	0.85	<0.001	2.33	1.71	3.18
Community media	No	1.38	<0.001	3.99	3.04	5.25
Electronic media	No	0.59	<0.001	1.80	1.32	2.45
Education	<7 years		0.002			
7–9 years study		0.15	0.335	1.16	0.86	1.56
>9 years study		0.71	0.001	2.04	1.36	3.05
Constants		−2.51	<0.001	0.08		

Among all the above significant factors, the degree of knowledge regarding malaria of villagers who received malaria-related information through community media was 3.99 times higher than those who did not (OR 3.99, 95 % CI 3.04–5.25, p < 0.001). The degree of knowledge regarding malaria of residents aged below 60 were 3.38 times higher than those of villagers were below 29 years old (OR 3.38, 95 % CI 2.20–5.19, p < 0.001). Moreover, the degree of knowledge regarding malaria of participants in type-1 and type-2 counties were higher than those in type-3 counties (OR 1.61, 95 % CI 1.14–2.29, p = 0.007; OR 2.63, 95 % CI 1.94–3.56, p < 0.001, respectively). The degree of knowledge regarding malaria of villagers who had more than 9 years study was 2.04 greater than those of who had less than 7 years study (OR 2.04, 95 % CI 1.36–3.05, p = 0.001). Interestingly, compared with residents living within 1 km to township hospital, the degree of knowledge regarding malaria of them seemed to decrease alongside the increase in distance. At last, whether one received malaria knowledge through electronic media and a history of suffering from malaria was clearly associated with their awareness of malaria (OR 1.80, 95 % CI 1.32–2.45, p < 0.001; OR 2.33, 95 % CI 1.71–3.18, p < 0.001, respectively).

### Determinants of malaria awareness of the sample population

After identifying the association between the rates of high-degree-knowledge and the group distribution rate, Cochrane and Mantel–Haenszel test was applied to assess their interaction. Table 3 shows the comparison of the rates of high-degree-knowledge among hierarchical subgroups. Almost in all subgroups (except the covariates of worker and high income family), villagers who had received malaria information through community media had higher rate of achieving high-level of knowledge than those who had not. However, a consistent effect of community media across various subgroups were showed through this observed cross-sectional study.

**Table 3:** Malaria knowledge status between participants within and beyond the coverage of community media

Characteristics	Beyond the coverage of community media		Within the coverage of community media		OR	95 % CI	
	No. with high degree of malaria knowledge/total no.	%	No. with high degree of malaria knowledge/total no.	%		Lower	Upper
Sex							
Male	191/413	46.2	165/230	71.7	2.95	2.09	4.17
Female	184/463	39.7	147/215	68.4	3.28	2.33	4.62
Ethnicity							
Han	272/589	46.2	202/276	73.2	3.18	2.33	4.34
Minorities	103/287	35.9	110/169	65.1	3.33	2.24	4.96
Age							
<29	57/184	31.0	62/104	59.6	3.29	1.99	5.43
30–44	89/207	43.0	77/118	65.3	2.49	1.56	3.98
45–59	109/250	43.6	115/144	79.9	5.13	3.18	8.27
>60	120/235	51.1	58/79	73.4	2.65	1.51	4.64
Education attainment							
<7 years	166/397	41.8	117/169	69.2	3.13	2.14	4.59
7–9 years study	140/334	41.9	123/183	67.2	2.84	1.95	4.14
>9 years study	69/145	47.6	72/93	77.4	3.78	2.10	6.78
Type of occupation							
Self-employed worker	36/135	46.7	57/84	67.9	2.41	1.37	4.26
Farmer migrant	224/515	43.5	192/263	73.0	3.51	2.54	4.85
Non-work	50/142	35.2	31/51	60.8	2.85	1.48	5.51
Worker	22/38	57.9	11/14	78.6	2.67	0.64	11.14
Students	16/46	34.8	21/33	63.6	3.28	1.29	8.34
Number of household members							
<3	143/269	53.2	81/122	66.4	1.74	1.11	2.72
3–4	180/428	42.1	175/239	73.2	3.77	2.67	5.32
>4	45/155	29.0	47/66	71.2	6.05	3.20	11.42
Marital status							
No	40/112	35.7	44/67	65.7	3.44	1.82	6.50
Yes	273/640	42.7	253/348	72.7	3.58	2.70	4.75
Type of county							
Type-1	132/263	50.2	97/141	68.8	2.19	1.42	3.36
Type-2	174/345	50.4	131/170	77.1	3.30	2.18	5.00
Type-3	69/268	25.7	84/134	62.7	4.85	3.11	7.56
Self-assessment of household income level							
Low	134/372	36.0	123/181	68.0	3.77	2.58	5.49
Middle	202/416	48.6	163/215	75.8	3.32	2.30	4.79
High	33/77	42.9	21/44	47.7	1.22	0.58	2.56
Distance to township hospital (km)							
<1	109/222	49.1	85/119	71.4	2.59	1.61	4.18
1–2	65/187	34.8	82/119	68.9	4.16	2.55	6.80
2–3	54/143	37.8	54/80	67.5	3.42	1.92	6.10
>3	97/259	37.5	87/123	70.7	4.04	2.54	6.41

## Discussion

Prior studies were reported at the beginning of NMEP, however, the determinants of malaria knowledge have not been explored [24, 31]. Under such circumstances, the purpose of this study was set to explore the predictors of public awareness of malaria at the transition period stage of the NMEP program across different MECs of China. According to the main findings from the collected data, more than half of villagers had high degree of malaria knowledge, and the patterns of publicizing malaria knowledge through community media affected the public malaria awareness the most.

It may have benefited from different forms of publicity materials. The community media publicizing malaria knowledge was different in various counties. In general, health information including malaria knowledge were delivered to residents through bulletin boards of village clinics and township hospitals periodically, which similar positive effect to the knowledge, attitude, and behaviour of rural residents were also found in rural China [32, 33]. In addition, according to an informed source in this investigation, as a work task, health sectors are required to publish information regarding malaria in newspaper monthly to consolidate the achievements of malaria elimination, and to increase public awareness of malaria.

It may also have benefited from the activities, which were conducted to cover different target population. In health education and promotion activities, exercise books with basic malaria knowledge as common publicity materials were provided to students. Shopping bags, aprons and disposable cups carrying information of malaria were distributed to the public in some MECs. Leaflets, banner advertisements were also used to spread malaria knowledge to the public and workers who are migrating to malaria-endemic regions.

Although only 33.7 % of the villagers received malaria knowledge through community media. However, it indicates this effective publicity material should be implemented in a larger range and strengthen this publicity effort to cover more residents, especially the target population. Additionally, given that most of malaria infections are originated in Africa and Asia [23], publicity may worth performing in the international collaboration of health education among at-risk population [34].

There were significant associations between the endemic type of county, experience of suffering malaria, self-assessed household income level, educational attainment and the degree of knowledge regarding malaria. The residents who received more than 9 years of education, with middle household income, living in type-1 and type-2 counties, having received malaria knowledge from electronic and with history of suffering malaria had high degree of malaria knowledge in this study. It indicates that the authorities should pay attention to the population who received less than 9 years of education, with lower household income, living in type-3 malaria counties, and those who did not received any malaria information in remote areas.

Interestingly, the rates of high-degree-knowledge of malaria were higher among the aged population, and those living in shorter distance to township hospitals. A possible explanation for this trend may be due to the fact that the middle aged and the elderly have experienced the outbreaks of malaria. The older the age, the greater the possibility of suffering from malaria, and the more sufficient knowledge of malaria they may have obtained. In addition, as shown in Table 4, the rates of high-degree-knowledge due to a history of suffering from malaria in different types of MECs substantially increased with age.

**Table 4:** The rates of high degree of malaria knowledge due to a history of suffering from malaria in different types of malaria endemic counties

Age	Type 1		Type 2		Type 3		Total	
	n/N	%	n/N	%	n/N	%	n/N	%
<29	9/28	32.14	6/54	9.38	1/27	3.70	16/119	13.45
30–44	7/54	12.96	18/84	21.43	3/28	10.71	28/166	16.88
45–59	37/90	41.11	29/80	36.25	12/54	22.22	78/224	34.82
>60	27/57	47.37	38/77	49.35	12/44	27.27	77/178	43.26

*n* is the number of high degree of malaria knowledge due to history of suffering from malaria. *N* is the total number of high degree of malaria knowledge

The trend of malaria knowledge increased with the distances to township hospitals declined, which may also be explained by the fact that the publicity work in rural areas was mainly dependent on the health workers in township hospitals, and its coverage effect was naturally raised with the shorter distance to township hospitals. It was worth noting that the youth and the residents who lived far away from township hospitals tended to have a lower-level of knowledge regarding malaria, and thus influencing the effect of the malaria education campaign. Therefore, the malaria promotion campaigns should focus especially on the residents in remote areas.

## Limitations

This study has several limitations. The cross-sectional study was only investigated the villages from nine MECs, whereas Hainan and Yunan provinces, which were considered as classical malaria endemic religions in China, were not sampled. Consequently, the results might be unrepresented due to the sample population. The dependent variables were measured through close-ended questions. It might allow the respondents to guess the correct answers to some extent. However, it still provided reference basis for the further design of in-depth research to develop malaria educational materials and improve the malaria knowledge promotion in the next phase of the NMEP.

## Conclusions

Malaria knowledge promotion is an integral part of the national malaria elimination campaign. This study intends to explore the associations between the public malaria awareness and their socio-demographic characteristics as well as sources of malaria information. Community media (including bulletin boards of village clinics or township hospitals, newspapers, exercise books, shopping bags, aprons, disposable cups, leaflets, and banner advertisements) played the most important role in influencing public awareness of malaria in the sample population, nevertheless, only few respondents received malaria information through this media. Moreover, age, distance to township hospitals, endemic county type, a history of suffering from malaria, electronic media, self-assessment of household income level and education were also significantly associated with public awareness of malaria. This suggests that community media was effective publicity material, which should be implemented to a larger extent. Malaria education campaign need to be aligned with target populations, in particular, people who are under 45 years old and residents (especially in type-3 counties) in remote areas.

## Abbreviations

WHO: World Health Organization
NMEP: national malaria elimination programme
MEC: malaria endemic county
OR: odds ratio
CI: confidence interval

## References

[1] WHO. World Malaria Report 2015. Geneva: World Health Organization. 2015. http://www.who.int/malaria/publications/world-malaria-report-2015/en/.

[2] Moonen B, Cohen JM, Snow RW, Slutsker L, Drakeley C, Smith DL (2010). Operational strategies to achieve and maintain malaria elimination. Lancet.

[3] Noor AM, Kinyoki DK, Mundia CW, Kabaria CW, Mutua JW, Alegana VA (2014). The changing risk of *Plasmodium falciparum* malaria infection in Africa: 2000–10: a spatial and temporal analysis of transmission intensity. Lancet.

[4] WHO. Global Technical strategy for malaria 2016–2030. Geneva: World Health Organization. http://www.who.int/malaria/areas/global_technical_strategy/en/.

[5] The Asia pacific malaria elimination network. Elimination 2030. http://apmen.org/elimination2030/.

[6] Yin JH, Zhou SS, Xia ZG, Wang RB, Qian YJ, Yang WZ (2014). Historical patterns of malaria transmission in China. Adv Parasitol.

[7] Feng J, Xiao H, Xia Z, Zhang L, Xiao N (2015). Analysis of malaria epidemiological characteristics in the People’s Republic of China, 2004–2013. Am J Trop Med Hyg.

[8] Zhang Q, Lai S, Zheng C, Zhang H, Zhou S, Hu W (2014). The epidemiology of *Plasmodium vivax* and *Plasmodium falciparum* malaria in China, 2004–2012: from intensified control to elimination. Malar J.

[9] National health and family planning commission of the People’s Republic of China. Action Plan of China Malaria Elimination (2010–2020) (in Chinese). http://www.gov.cn/gzdt/att/att/site1/20100526/001e3741a2cc0d67233801.doc.

[10] Hu T, Liu YB, Zhang SS, Xia ZG, Zhou SS, Yan J (2016). Shrinking the malaria map in China: measuring the progress of the national malaria elimination programme. Infect Dis Poverty.

[11] Tatem AJ, Smith DL, Gething PW, Kabaria CW, Snow RW, Hay SI (2010). Ranking of elimination feasibility between malaria-endemic countries. Lancet.

[12] Li Z, Zhang Q, Zheng C, Zhou S, Sun J, Zhang Z (2016). Epidemiologic features of overseas imported malaria in the People’s Republic of China. Malar J.

[13] Cohen JM, Smith DL, Cotter C, Ward A, Yamey G, Sabot OJ (2012). Malaria resurgence: a systematic review and assessment of its causes. Malar J.

[14] Abeyasinghe RR, Galappaththy GN, Smith GC, Kahn JG, Feachem RG (2012). Malaria control and elimination in Sri Lanka: documenting progress and success factors in a conflict setting. PloS ONE.

[15] Andriopoulos P, Economopoulou A, Spanakos G, Assimakopoulos G (2013). A local outbreak of autochthonous *Plasmodium vivax* malaria in Laconia, Greece–are-emerging infection in the southern borders of Europe?. Int J Infect Dis.

[16] Tanner M, Vlassoff C (1998). Treatment-seeking behaviour for malaria: a typology based on endemicity and gender. Soc Sci Med.

[17] Cropley L (2004). The effect of health education interventions on child malaria treatment-seeking practices among mothers in rural refugee villages in Belize, Central America. Health Promot Int.

[18] Cao J, Sturrock HJW, Cotter C, Zhou S, Zhou H, Liu Y (2014). Communicating and monitoring surveillance and response activities for malaria elimination: China’s “1-3-7″ strategy. PLos Med.

[19] Zhou S, Zhang S, Zhang L, Rietveld AEC, Ramsay AR, Zachariah R (2015). China’s 1-3-7 surveillance and response strategy for malaria elimination: is case reporting, investigation and foci response happening according to plan?. Infect Dis Poverty.

[20] Das A, Das GR, Friedman J, Pradhan MM, Mohapatra CC, Sandhibigraha D (2013). Community perceptions on malaria and care-seeking practices in endemic Indian settings: policy implications for the malaria control programme. Malar J.

[21] Liu H, Li M, Jin M, Jing F, Wang H, Chen K (2013). Public awareness of three major infectious diseases in rural Zhejiang province, China: a cross-sectional study. BMC Infect Dis.

[22] Hlongwana KW, Mabaso ML, Kunene S, Govender D, Maharaj R (2009). Community knowledge, attitudes and practices (KAP) on malaria in Swaziland: a country earmarked for malaria elimination. Malar J.

[23] Qian YJ, Zhang L, Xia ZG, Vong S, Yang WZ, Wang DQ (2014). Preparation for malaria resurgence in China: approach in risk assessment and rapid response. Adv Parasitol.

[24] Yin J, Xia Z, Wang R, Zhang Q, Fang W, Zhou S (2015). Public awareness of malaria at the beginning of a national malaria elimination program in China. J Infect Dev Ctries.

[25] Fu H, Hu T, Wang J, Feng D, Fang H, Wang M (2015). A bibliometric analysis of malaria research in China during 2004–2014. Malar J.

[26] Tang SF, Wang X, Zhang Y, Hou J, Ji L, Wang ML (2015). Analysis of high alert medication knowledge of medical staff in Tianjin: a convenient sampling survey in China. J Huazhong Univ Sci Technolog Med Sci.

[27] Yuan F, Qian D, Huang C, Tian M, Xiang Y, He Z (2015). Analysis of awareness of health knowledge among rural residents in Western China. BMC Public Health.

[28] Tian M, Chen Y, Zhao R, Chen L, Chen X, Feng D (2011). Chronic disease knowledge and its determinants among chronically ill adults in rural areas of Shanxi Province in China: a cross-sectional study. BMC Public Health.

[29] Sun X, Feng Z, Zhang P, Shen X, Wei L, Tian M (2014). Association between time of pay-for-performance for patients and community health services use by chronic patients. PloS ONE.

[30] Tang S, Bishwajit G, Ji L, Feng D, Fang H, Fu H (2016). Improving the blood pressure control with the proactive attitude of hypertensive patients seeking follow-up services: evidence from China. Med.

[31] Yin J, Wang R, Xia Z, Zhou S, Zhou X, Zhang Q (2013). Students’ awareness of malaria at the beginning of national malaria elimination programme in China. Malar J.

[32] Sun X, Chen Y, Tong X, Feng Z, Wei L, Zhou D (2014). The use of annual physical examinations among the elderly in rural China: a cross-sectional study. BMC Health Serv Res.

[33] Tang S, Dong D, Ji L, Fu H, Feng Z, Bishwajit G (2015). What contributes to the activeness of ethnic minority patients with chronic illnesses seeking allied health services? a cross-sectional study in rural Western China. Int J Environ Res Public Health.

[34] Xia ZG, Wang RB, Wang DQ, Feng J, Zheng Q, Deng CS (2014). China-Africa cooperation initiatives in malaria control and elimination. Adv Parasitol.

